# Rehabilitation of Ectodermal Dysplasia Using CAD/CAM Mandibular Complete Denture and Maxillary Overdenture: A Clinical Report

**DOI:** 10.1155/2024/9705699

**Published:** 2024-01-27

**Authors:** Hatem Alqarni, Faisal Alzeghaibi, Sahar Alotaibi, Raghad Alamri, Raghad Aljohani, Majed S. Altoman, Mohammed A. Alfaifi

**Affiliations:** ^1^Department of Restorative and Prosthetic Dental Sciences, College of Dentistry, King Saud bin Abdulaziz University for Health Sciences, Riyadh, Saudi Arabia; ^2^King Abdullah International Medical Research Center, Ministry of National Guard Health Affairs, Riyadh, Saudi Arabia; ^3^College of Dentistry, King Saud bin Abdulaziz University for Health Sciences, Riyadh, Saudi Arabia; ^4^Department of Prosthetic Dental Sciences, King Khalid University College of Dentistry, Abha, Saudi Arabia

## Abstract

Ectodermal dysplasia is a genetic disorder characterized by the abnormal development of two or more ectodermally driven structures, leading to various clinical manifestations such as sparse hair, dry skin, and hypodontia or anodontia. The absence of teeth significantly impacts the quality of life for individuals affected by this condition. This article presents a clinical case report of a patient with ectodermal dysplasia who underwent full mouth rehabilitation using computer-aided design/computer-aided manufacturing (CAD/CAM) technology to fabricate a mandibular complete denture and a maxillary overdenture.

## 1. Introduction

Ectodermal dysplasia (ED) encompasses a heterogeneous collection of genetic disorders that manifest as abnormalities in two or more tissues derived from the ectodermal germ layer, including the skin, hair, teeth, nails, and sweat glands. Affected individuals typically exhibit hypotrichosis, characterized by the scarce distribution of hair on the scalp and body, as well as hypohidrosis, which entails a diminished capacity to perspire. Furthermore, they commonly present with hypodontia, a condition marked by the congenital absence of teeth [1]. The prevalence of hypohidrotic ectodermal dysplasia (ED) within the general population has been approximated to range from 1 in 10,000 to 1 in 100,000 male live births [2].

Dental anomalies associated with ectodermal dysplasia encompass a spectrum ranging from complete absence of teeth (anodontia) to a less severe form known as hypodontia, which is more commonly observed [3]. The condition affects both the primary and permanent dentition, with conical or peg-shaped teeth being the prevalent characteristics [3]. In cases where teeth are entirely absent, the alveolar process, which supports the teeth, undergoes inadequate development. Consequently, the occlusal vertical dimension (OVD) is diminished, leading to a facial appearance resembling that of a patient with edentulism. Despite these dental abnormalities, it is important to note that the growth and overall development of affected children remain unaffected [4, 5]. Early initiation of dental treatment is crucial in the management of patients with ectodermal dysplasia. This proactive approach is motivated by several primary considerations, including functional, psychological, and esthetic aspects. Addressing dental issues at an early stage is aimed at optimizing oral function, enhancing psychological well-being, and improving overall esthetics [6, 7]. The facial appearance resulting from dental abnormalities in ectodermal dysplasia can give rise to an unfavorable self-image, leading to social withdrawal and challenges in the individual's social integration within society [7–9]. The dental appearance of young patients affected by ectodermal dysplasia holds significant significance [7–9]. Therefore, determining the timing and sequence of treatment should involve collaborative decision-making with the patient's parents. The treatment options may encompass fixed, removable, or implant-supported fixed partial dentures [7–9]. Removable prosthodontics stands as the predominant treatment modality employed in addressing ectodermal dysplasia (ED) [10]. In cases where teeth provide adequate support, overdentures have demonstrated successful outcomes. Notably, removable prosthodontics offers several advantages, including its simplicity, cost-effectiveness, and reversibility. Moreover, this conservative approach preserves the natural dentition of young patients and helps maintain the integrity of the alveolar bone [10–12]. While implants are an excellent treatment option for adults with ED, it is important to consider the limitation of cost associated with this approach. The comprehensive nature of implant therapy, including surgical procedures, prosthetic components, and long-term maintenance, can result in substantial financial implications [13].

The development of computer-aided design and computer-aided manufacturing (CAD/CAM) systems has brought significant advancements in the fabrication of removable complete dentures (RCDs) [14]. When compared to conventionally processed dentures, CAD-CAM RCDs exhibit several notable advantages [15]. These include a reduction in residual monomer content, improved physical properties of the acrylic resin base, decreased polymerization shrinkage, and diminished adhesion of Candida albicans organisms to the denture base. Furthermore, CAD-CAM RCDs can be provided with a reduced number of patient visits, and the use of digital files allows for easier remake when necessary [15]. Moreover, denture base adaptation and retention are notably improved in CAD-CAM RCDs [16]. These advancements in digital dentistry have significantly contributed to enhancing the quality, efficiency, and patient experience in the fabrication of removable complete dental prostheses [17, 18].

The progress of digital dentistry has had a positive impact on the quality of treatment for removable dentures by streamlining the design and fabrication processes [18, 19]. Notably, computer-engineered complete dentures (CECD) have demonstrated superior retention and adaptation compared to conventional methods. This advancement in technology has simplified the workflow and allowed for more precise and efficient production of complete dentures, resulting in improved outcomes for patients [20, 21].

The present clinical report outlines a novel approach employed for an adolescent patient diagnosed with ectodermal dysplasia. The therapeutic intervention involved the utilization of a monolithic mandibular complete denture and maxillary overdenture, both created using a digital workflow facilitated by computer-aided design and computer-aided manufacturing (CAD/CAM) technology.

## 2. Case Report

**Figure 1:**
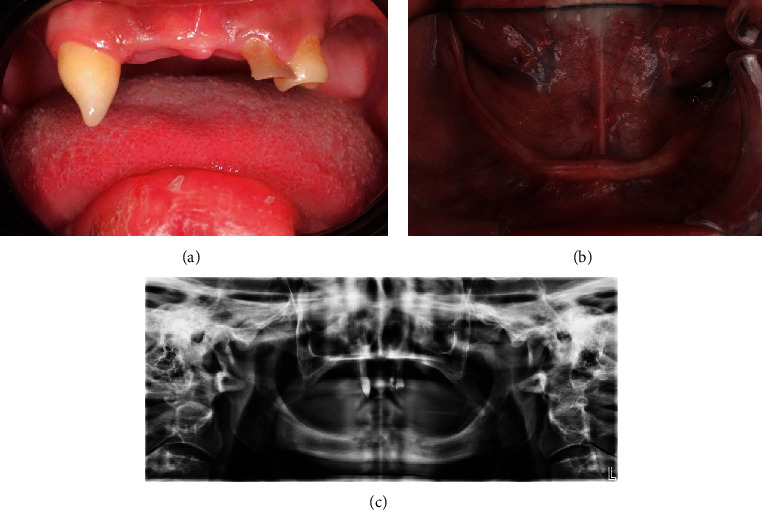
(a) Initial presentation maxillary arch. (b) Initial presentation mandibular arch. (c) Panoramic radiograph.

A 23-year-old male presented to the dental clinics at King Saud bin Abdulaziz University, Riyadh, Saudi Arabia, with a primary concern of restoring his teeth, improving his speech, and restoring his function (Figure 1(a)). He did not disclose any habits, and his medical history was insignificant. The patient had anomalies in his skin, hair, and nails when they first showed up. The craniofacial characteristics encompassed a concave nasal bridge, diminished vertical facial dimension in the lower third, prominent mentum, profound nasolabial furrow, and conspicuous frontal prominence. In the intraoral domain, the individual exhibited deficient tooth development in the upper maxillary arch, complete absence of teeth in the lower arch, and hypertrophy of the tongue (a manifestation of acromegaly). A comprehensive and meticulous clinical appraisal led to the provisional diagnosis of ED. The comprehensive intraoral examination revealed a severely decayed remaining maxillary lateral incisor, peg-shaped rotated canine. Supplementary to these observations, the patient displayed indications of xerostomia, a prevalent characteristic within the spectrum of manifestations observed in individuals afflicted by ED. The shape of the residual alveolar ridges was square in the maxillary arch and U-shaped in the mandibular arch (Figure 1(b)). Radiographic examination revealed no abnormalities other than severely resorbed maxillary and mandibular alveolar ridges (Figure 1(c)).

**Figure 2:**
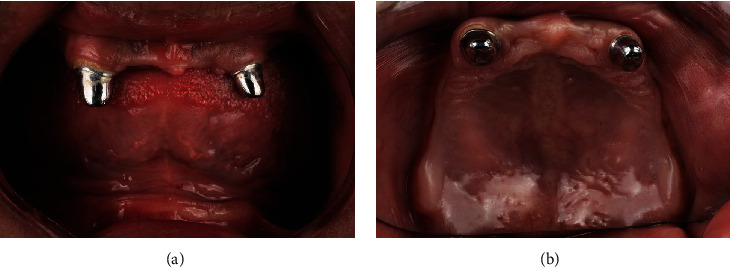
Metal copings after cementation: (a) frontal view; (b) occlusal view.

**Figure 3:**
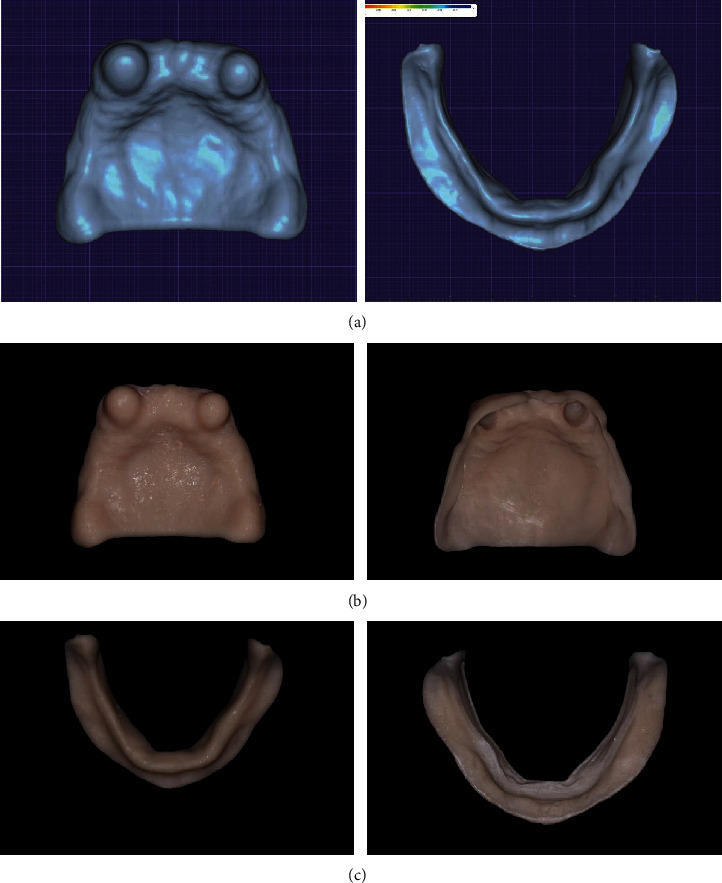
(a) Design of the record base. (b) Maxillary printed record base. (c) Mandibular printed record base.

**Figure 4:**
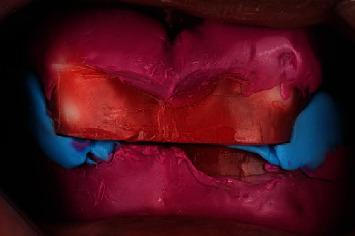
Maxillomandibular relationship record.

**Figure 5:**
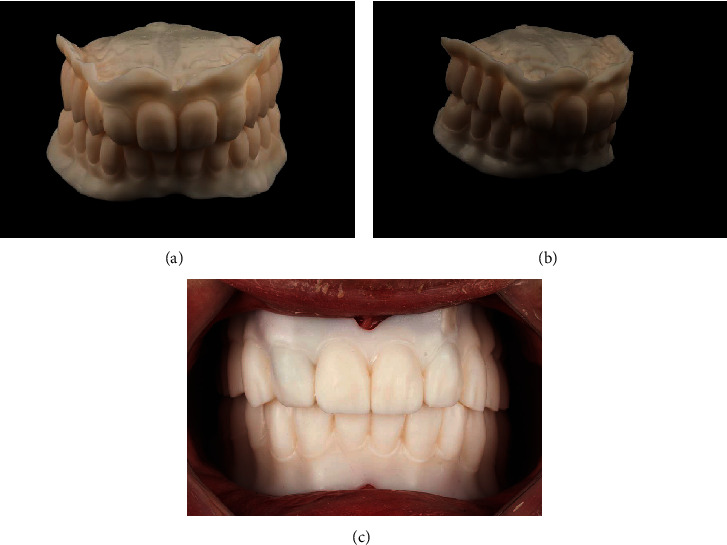
Printed denture prototype: (a) frontal view; (b) lateral view; (c) intraoral try-in.

**Figure 6:**
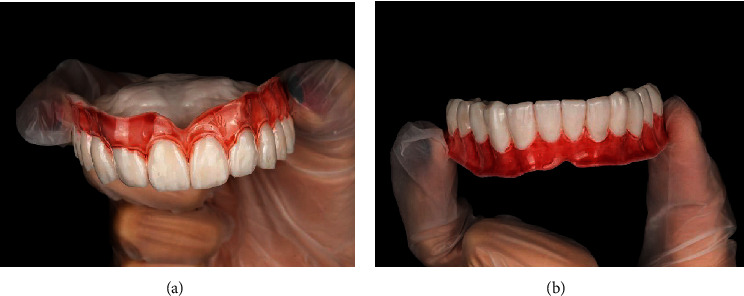
Printed final prosthesis: (a) maxillary overdenture; (b) mandibular complete denture.

**Figure 7:**
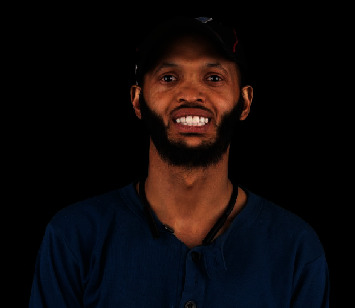
Extraoral frontal view of final prosthesis.

The patient was presented with various options of conventional removable prostheses, implant-retained removable prostheses, or fixed prostheses. The patient decided on a removable prosthesis due to his limited financial means. The definitive treatment plan was for a CAD/CAM 3-dimensionally printed mandibular complete denture and maxillary overdenture. The first treatment phase included extraction of the severely decayed maxillary lateral incisor. The peg-shaped rotated maxillary canines underwent intentional endodontic therapy, and then, full-metal copings were placed which were used as an overdenture attachment. The metal copings were cemented using resin cement (3M RelyX Unicem Self-Adhesive Universal Resin Cement) (Figures 2(a) and 2(b)). After complete healing of three months of the extraction socket, a preliminary impression was made using irreversible hydrocolloid impressions (Palgat Plus Quick; 3M ESPE, USA). The preliminary diagnostic casts were scanned using an intraoral scanner (TRIOS; 3Shape) and imported into a computer-aided design and computer-aided manufacturing software program (DentalCAD® 3.1, Rijeka) where record bases were fabricated and 3D printed using a PMMA resin material (Freeprint matrix; Detax, GmbH Carl-Zeiss-Str.4) (Figures 3(a)–3(c)). The adaptation of the record bases was verified intraorally, and no necessary corrections were needed. During this visit, maxillomandibular relation registration was done using registration bases (Freeprint matrix; Detax, GmbH), occlusal rims (Hygienic medium-soft no. 3 pink wax; Coltene) and bite registration material (Vanilla Bite™; US). On the same visit, border molding and definitive impressions were made using polyvinyl siloxane (Imprint 4; 3M ESPE) to record the tissues in functional form (Figure 4). Then, an extraoral scan of the cameo and intaglio surfaces of the maxillomandibular recordings was performed using a laboratory scanner (D1000; 3Shape, Copenhagen, Denmark). The maxillomandibular relationship standard tessellation language (STL) files were imported into DentalCAD® 3.1 (Rijeka) software for mounting on a virtual articulator. This file was then used to digitally set up and design a denture prototype, which was 3-dimensionally printed using a PMMA resin (Freeprint matrix; TRYIN, GmbH) (Figure 5). Afterward, the printed try-in prototype was placed to evaluate the phonetics, esthetics, and function of the denture. These interim restorations required chair-side modifications including correction of occlusal interference. Once the modifications had been completed, a wash impression with polyvinyl siloxane (Imprint 4; 3M ESPE) was made since the prototype denture base was underextended (Figures 5(a)–5(c)). The prototype was scanned using a laboratory scanner (D1000; 3Shape, Copenhagen, Denmark) and used as a definitive impression of the tissue surface. With this design approved, the prosthesis was sent back to the laboratory for the required changes to be replicated in the final restoration. The design was 3-dimensionally printed using a PMMA resin (Freeprint matrix; Detax, GmbH) (Figures 6(a) and 6(b)). In the placement appointment, pressure indicator paste (PIP) (PSI Coltene) was used to evaluate the fit of the intaglio surface to the mucosa. Esthetics, phonetics, and occlusion were then evaluated, then dentures were delivered, and postoperative was explained to ensure proper oral hygiene (Figure 7). The patient was scheduled for follow-up for intervention, and if necessary, adjustments were needed.

## 3. Discussion

A preliminary diagnosis of ED was formulated through an assessment of the following clinical manifestations: notably reduced hair density encompassing the eyebrows and eyelashes, accompanied by parched skin exhibiting periorbital hyperkeratosis; discernible dysmorphic facial characteristics; compromised exocrine gland activity; and the presence of a familial ED lineage [1]. Timely dental intervention for ectodermal dysplasia (ED) holds significant relevance in enhancing the overall quality of life for young patients. This approach yields substantial enhancements across domains encompassing functional aspects, psychological well-being, and esthetic considerations [6, 7].

The current protocol effectively combines the benefits of the digital workflow in CAD/CAM dentures and conventional maxillomandibular recordings. The workflow is completely driven by CAD/CAM technology and eliminates many of the disadvantages of laboratory steps required by traditional denture fabrication. One of the limitations of this case report is that long-term follow-ups are not available. An established protocol for the prosthodontic treatment approach and workflow in patients with ED is lacking. However, a successful treatment plan should consider the patient's age, skeletal development, minimally invasive protocols, and treatment durability. Further clinical reports and research are needed to validate the fully digital workflow since digital dentistry has been improved and applied in many dental aspects.

## 4. Conclusion

This clinical report described a digital workflow to complete oral rehabilitation of a patient diagnosed with ED by using a digital workflow. The thorough and interdisciplinary approach supported by digital technologies was directly related to the favorable and accurate treatment outcome.
